# Fructose-1,6-Bisphosphatase 2 Inhibits Oral Squamous Cell Carcinoma Tumorigenesis and Glucose Metabolism via Downregulation of c-Myc

**DOI:** 10.1155/2022/6766787

**Published:** 2022-05-06

**Authors:** Liang Wang, Jinbing Wang, Yi Shen, Zhiwei Zheng, Jian Sun

**Affiliations:** Department of Oral Maxillofacial-Head Neck Oncology, Shanghai Ninth People's Hospital, Shanghai Jiao Tong University School of Medicine, National Clinical Research Center for Oral Diseases, Shanghai Key Laboratory of Stomatology & Shanghai Research Institute of Stomatology, Shanghai 200011, China

## Abstract

**Background:**

Fructose-1,6-bisphosphatase 2 (FBP2), known as a rate-limiting enzyme in gluconeogenesis, is a tumor suppressor downregulated in various cancers. However, the role of FBP2 in oral squamous cell carcinoma (OSCC) remains largely unclear.

**Methods:**

The level of FBP2 in OSCC tissues and matched adjacent normal tissues was determined by western blot and RT-qPCR assays. In addition, analysis of FBP2 function in OSCC cells was assessed using both gain-of-function and loss-of-function studies.

**Results:**

In this study, we found that the expression of FBP2 was remarkably downregulated in OSCC tissues and OSCC cells. Overexpression of FBP2 suppressed the viability, proliferation, migration, and glycolysis of OSCC cells, whereas FBP2 knockdown exhibited the opposite results. Moreover, downregulation of FBP2 promoted the growth and glycolysis of OSCC cells in nude mice in a xenograft model. Specifically, FBP2 colocalizes with the c-Myc transcription factor in the nucleus. Significantly, inhibitory effects of FBP2 overexpression on the viability, proliferation, migration, and glycolysis of OSCC cells were reversed by c-Myc overexpression.

**Conclusion:**

Collectively, FBP2 could suppress the proliferation, migration and glycolysis in OSCC cells through downregulation of c-Myc. Our study revealed a FBP2-c-Myc signaling axis that regulates OSCC glycolysis and may provide a potential intervention strategy for OSCC treatment.

## 1. Introduction

Oral squamous cell carcinoma (OSCC) has been considered to be the most prevalent malignancy in the head and neck region [1, 2]. In addition, OSCC has a tendency for local invasion and a potential for cervical lymph node metastasis [3–5]. Moreover, OSCC often leads to oral dysfunction including chewing and swallowing disorders, which profoundly worsens a patient's quality of life [6]. Recently, surgery, chemotherapy, radiotherapy, and adjuvant chemoradiotherapy are the main treatment strategies for OSCC treatment [7–9]. Despite substantial progress in the diagnosis and treatment of OSCC, the 5-year overall survival rates of OSCC remain no more than 50% [6, 10]. Therefore, discovery effective biomarkers and therapeutic targets may help to understand the prognosis and treatment of OSCC.

One remarkable feature of cancer is the reprogramming energy metabolism [11]. Glucose metabolism is a major type of energy metabolism [12]. Under normal conditions, cells generate energy on the process of aerobic respiration [12]. Meanwhile, cells use glycolysis to generate energy when the oxygen is not enough [12]. As early as the 1950s, Otto Warburg performed a study of cancer metabolism and discovered that cancer cells preferentially metabolize glucose through aerobic glycolysis whether there is sufficient oxygen present [13, 14]. Glycolysis plays a key role in the growth and metastasis of human cancers [15, 16]. Malignant tumors, including OSCC, rely on aerobic glycolysis to maintain rapid cell growth [17].

Fructose-1,6-bisphosphatase 2 (FBP2) is a rate-limiting enzyme in gluconeogenesis [18]. Gluconeogenesis, a reverse process of glycolysis, is important for the maintenance blood glucose levels during starvation [19]. Li et al. found that FBP1 loss could promote glycolysis and cell growth of gastric cancer cells [20]. Huangyang et al. showed that FBP2 restoration could suppress glycolysis activity in sarcoma [18]. However, the role of FBP2 in OSCC remains largely unclear. On the other hand, c-Myc was known to be a crucial mediator in tumor progression [21, 22]. In addition, c-Myc could act as a key modulator in the regulation of glucose metabolism through mediation of GLUT1, HK2, PKM2, and LDHA [23]. However, the relation between FBP2 and c-Myc in OSCC remains unexplored.

Thus, this study aimed to investigate the function of FBP2 in glycolysis, proliferation, and migration in OSCC.

## 2. Materials and Methods

### 2.1. Clinical Samples

A total of 10 paired OSCC tissues and matched adjacent normal tissues were obtained from the Shanghai Ninth People's Hospital, Shanghai Jiao Tong University School of Medicine between April 2019 and September 2020. This study was approved by the ethics committee of Shanghai Ninth People's Hospital, Shanghai Jiao Tong University School of Medicine. The written consent was obtained from each patient.

### 2.2. RNA-Sequencing (RNA-seq) Analysis

Trizol reagent (Thermo Fisher Scientific) was used to extract total RNA from tissues. Sequencing libraries were prepared from all samples using the NEBNext Ultra Directional RNA Library Prep Kit for Illumina (NEB, Beverly, MA). After that, the libraries were quantified by Agilent Bioanalyzer 2100 and sequenced by Illumina Hiseq sequencer (Illumina, San Diego, CA, USA).

### 2.3. Microarray Data Analysis

GSE35261 dataset which contain the gene expression data of OSCC tissues and normal tissues was downloaded from GEO database. The differentially expressed genes (DEGs) between OSCC tissues and normal tissues were identified using the limma package.|log2 (fold change)|>1.0 with adjusted *P* value <0.05 was set as the screening threshold.

### 2.4. Intersected Analysis

The intersection of DEGs from in-house RNA-seq analysis (validation dataset) and GSE35261 dataset (testing dataset) was performed using the Venn diagram package.

### 2.5. KEGG Pathway Analyses

DEGs were evaluated by Kyoto Encyclopedia of Genes and Genomes (KEGG) enrichment analysis. Adjusted *P* < 0.05 was set as the threshold values.

### 2.6. Real-Time Quantitative Polymerase Chain Reaction (RT-qPCR)

The Trizol reagent (Invitrogen, USA) was used to extract total RNA from tissues and cells. Complementary DNA (cDNA) was synthesized using the ReverTra Ace qPCR RT Kit (TOYOBO, Japan). After that, the qPCR was performed on an Applied Biosystems™ 7500 Real-Time PCR System (Applied Biosystems, USA) with the Power SYBR Green PCR Master kit (Applied Biosystems, USA). RT-qPCRs were performed in triplicate. The protocol of amplification was as follows: 10 minutes at 95°C, followed by 40 cycles (15 s at 95°C and 60 s at 60°C). GAPDH works as an internal control. Primer sequences are listed in Table 1.

### 2.7. Western Blot Assay

BCA protein assay kit (Sangon Biotech, Shanghai, China) was used to measure the protein concentration. After that, the protein was separated by 10% SDS-PAGE and then transferred onto the polyvinylidene difluoride (PVDF) membranes. After blocking with 5% skim milk, the membranes were incubated overnight at 4°C with the following primary antibodies. Antibodies (Abcam Cambridge, MA, USA) were diluted to 1 : 1000 (GAPDH antibody was diluted to 1 : 6000) for betaine homocysteine S-methyltransferase (BHMT), gamma-glutamyl transpeptidase 6 (GGT6), Griffiths mental development scales (GMDS), fructose 1,6-bisphosphatase 1 (FBP1), fructose 1,6-bisphosphatase 2 (FBP2), carbonyl reductase [NADPH] 3 (CBR3), fucosyltransferase-3 (FUT3), cleaved caspase 3, BCL-XL, N-cadherin, E-cadherin, GLUT1, HK2, PKM2, LDHA, c-Myc, Ki67, and GAPDH. Later on, the membranes were incubated with the corresponding secondary antibodies (1 : 5000, Abcam) at room temperature for 1 h and then visualized using the enhanced chemiluminescence (Thermo Fisher Scientific).

### 2.8. Cell Culture

One nontumorigenic human oral epithelial cell line (MSK-Leuk1) and five OSCC cell lines HSC-3, SCC9, SCC25, HN4, and CAL-27 were obtained from Shanghai Zhong Qiao Xin Zhou Biotechnology Co., Ltd. (Shanghai, China). All cell lines were cultured in DMEM medium containing 10% FBS and incubated 37°C in a 5% CO_2_ incubator.

### 2.9. Cell Lentivirus Infection

Lentivirus-containing shRNA targeting FBP2 (FBP2 shRNA1, FBP2 shRNA2, and FBP2 shRNA3) and lentivirus-containing shRNA targeting c-Myc (c-Myc shRNA1) were purchased from GenePharma (Shanghai, China). The FBP2-overexpressing, c-Myc-overexpressing, and control lentiviruses were obtained from General Biol (Anhui, China) and called FBP2 OE, c-Myc OE, and OE NC, respectively. 293 T cells were transfected with indicated lentiviral plasmids for 72 h. Next, the supernatant containing lentivirus was filtered through a filter membrane (0.22-*μ*m pore size) and then transduced into HSC-3 and CAL-27 cells. Subsequently, the infected cells were selected by 2 *μ*g/mL of puromycin.

### 2.10. Cell Counting Kit 8 (CCK-8) Assay

HSC-3 and CAL-27 cells (2 × 10^4^ cells) were plated onto 96-well plates and incubated for 48 h. Next, 10 *μ*L of CCK8 reagent (Beyotime, Beijing, China) was added into each well, and cells were then incubated for 3 h. Finally, the absorbance values were detected by the automatic microplate reader (INFINITE M NANO, TECAN, Germany) at 450 nm.

### 2.11. Colony Formation Assay

HSC-3 and CAL-27 cells were seeded onto 6-well plates and incubated for 2 weeks. Later on, cells were fixed with 4% formaldehyde and then stained with 1% crystal violet for 15 min. Subsequently, cell colonies were observed and photographed by a microscope (Leica, German).

### 2.12. Wound-Healing Assay

HSC-3 and CAL-27 cells were plated into 6-well culture plates overnight at 37°C up to ~80% confluence. After that, cells were wounded with a sterile 20-*μ*L pipette tip. The wound closure was photographed with a microscope (ECLIPSE TS2, Nikon, Japan) at 0 h and 48 h.

### 2.13. Methylation-Specific PCR (MSP)

For MSP detection, a pair of primers for amplifying methylated CpG targets was obtained based on the CpG island sequence of the FBP2 promoter-proximal elements. The sequences were as follows: 5′-TTTTTATTTTTTTATTTCGGTGATC-3′ (forward) and 5′-GTATCTCCTCCCCTTTCTAACGTA-3′ (reverse). Other sequences of primers (performed to amplify unmethylated CpG targets) were as follows: 5′-TTATTTTTTTATTTTGGTGATTGT-3′ (forward) and 5′-CATATCTCCTCCCCTTTCTAACATA-3′ (reverse). MSP was performed via a standard PCR machine. PCR products were resolved by agarose gel electrophoresis and stained with EB.

### 2.14. Transwell Migration Assay

Cell migration was determined by transwell migration assay using transwell chambers (Corning, NY, USA). Briefly, HSC-3 and CAL-27 cells (1 × 10^4^ cells) suspended in serum-free medium were seeded on the upper transwell chamber. 600 *μ*L of DMEM containing 10% FBS was added into the bottom chamber. After 24 h of incubation, the migrated cells were stained with 0.1% crystal violet. Later on, migrating cells were observed and counted under a microscope (ECLIPSE TS2, Nikon, Japan).

### 2.15. Flow Cytometry Assay

The apoptotic HSC-3 and CAL-27 cells were examined by using the Annexin V-FITC apoptosis detection kit (Beyotime, China) on a flow cytometer (FACSCalibur, BD, USA).

### 2.16. Glucose Uptake Assay

Glucose uptake was measured in HSC-3 and CAL-27 cells using a glucose update assay kit (cat.no. ab136955, Abcam) following the manufacturer's procedure.

### 2.17. Measurement of Lactate and ATP

The D-lactate levels were measured in HSC-3 and CAL-27 cells using a D-lactate assay kit (cat.no. ab83429, Abcam) according to the manufacturer's procedure. The cellular ATP content was detected in HSC-3 and CAL-27 cells using an ATP colorimetric assay kit (cat.no. ab282930, Abcam) according to the manufacturer's procedure.

### 2.18. Animal Study

BALB/c nude mice (6–8 weeks old) were obtained from the SiPeiFu (Beijing) Biotechnology Co., Ltd. (Beijing, China). This study was approved by the Shanghai Ninth People's Hospital, Shanghai Jiao Tong University School of Medicine, and conducted according to the institutional guidelines. HSC-3 cells (2.5 × 10^6^) were injected subcutaneously into the left flank of each mouse. When the mean tumor volume reached 40 mm^3^, mice were divided randomly into four groups: control, shRNA NC, FBP2 shRNA1, and DDP groups. After that, 2.5 × 10^6^ HSC-3 or HSC-3 cells stably expressing shRNA NC, FBP2, and shRNA1 in 100 *μ*L PBS were injected into the left flank of nude mice. Mice in the DDP group were treated intraperitoneally (i.p.) with 2 mg/kg cisplatin (MedChemExpress, Shanghai, China) once every two days for 3 weeks. The tumor volume = (length x width^2^)/2. After 3 weeks, mice were sacrificed, and the tumors were isolated.

### 2.19. Coimmunoprecipitation (Co-IP) Assay

Co-IP was carried out using a Pierce™ Classic Magnetic IP/Co-IP Kit (Thermo Scientific, USA). Briefly, HSC-3 and CAL-27 cells were incubated with anti-FBP2 and anti-c-Myc antibodies overnight at 4°C. Later on, the samples were incubated with pierce protein A/G magnetic beads. Subsequently, the protein binding complex was subjected to western blot assay.

### 2.20. Immunofluorescence (IF) Analysis

HSC-3 and CAL-27 cells were seeded onto 24-well plates and fixed by 4% paraformaldehyde for 15 min and then permeabilized in 0.1% Triton X-100 for 5 min at room temperature. After that, cells were blocked with 1% BSA in PBS, and stained with FBP2 and c-Myc antibodies (Abcam) at 4°C overnight, and then incubated with the corresponding secondary antibody at room temperature for 1 h. The stained cells were observed using a fluorescence microscope (Leica, German).

### 2.21. Statistical Analysis

All data were repeated in triplicate. One-way analysis of variance (ANOVA) and Tukey's tests were carried out for multiple group comparisons. Values are shown as the mean ± SD. Differences were considered to be statistically significant at ∗*P* < 0.05.

## 3. Results

### 3.1. Identification of DEGs in the OSCC Tissues and Normal Tissues

**Figure 1:**
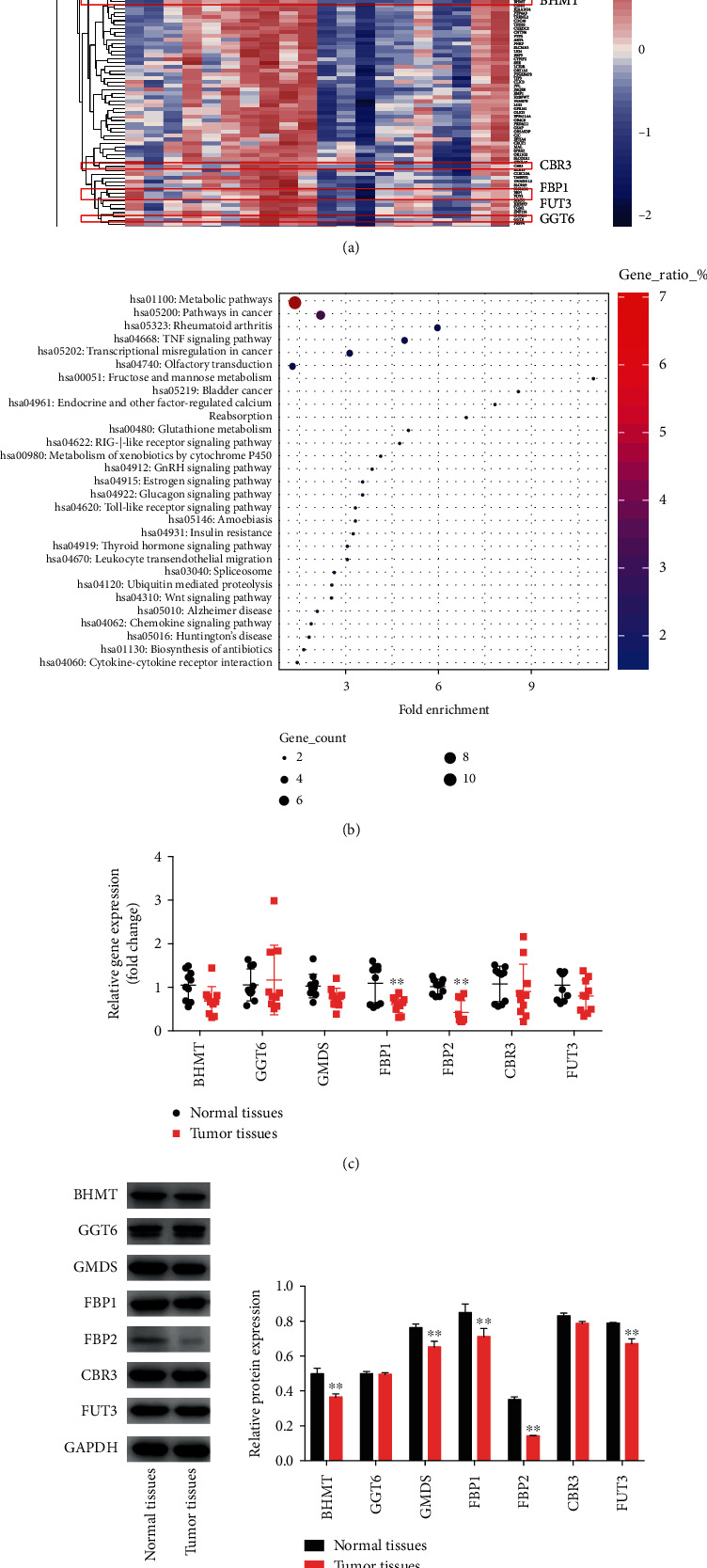
Identification of DEGs. (a) Heat maps of DEGs in in-house dataset (validation dataset). (b) DEGs were evaluated by KEGG pathway analysis. (c) RT-qPCR analysis of BHMT, GGT6, GMDS, FBP1, FBP2, CBR3, FUT3 levels in OSCC tissues and normal tissues. (d) BHMT, GGT6, GMDS, FBP1, FBP2, CBR3, and FUT3 levels in OSCC tissues and normal tissues were detected using western blot assay. ∗∗*P* < 0.01 vs. normal tissues group.

The DEGs between OSCC tissues and normal tissues were screened by GEO database (GSE35261, testing dataset) and validated by our in-house dataset (validation dataset). Our analysis found that a total of 777 DEGs were identified in the testing dataset and 193 DEGs were identified in the validation dataset (Supplementary Figures 1A, 1B, 2A, and 2B and Figure 1(a)). Meanwhile, 142 overlapping DEGs were identified in the testing dataset and validation dataset (Supplementary Figure 2C). For a more in-depth understanding of these overlapping DEGs, KEGG pathway enrichment analyses were performed. As revealed in Figure 1(b), these overlapping DEGs are mainly enriched in metabolic pathways. 10 overlapping genes (downregulated genes: BHMT, GGT6, GMDS, FBP1, FBP2, CBR3, FUT3; upregulated genes: PFKP, PLCB1, CYP3A5) participated in metabolic pathways (Figures 1(a) and 1(b)).

In addition, RT-qPCR assay confirmed that the mRNA levels of FBP1 (1.81-fold) and FBP2 (2.37-fold) were remarkedly reduced in OSCC tissues in comparison with the normal tissues (Figure 1(c)). The results of western blot assay showed that compared to normal tissues, OSCC tissues displayed significantly decreased expression of BHMT (1.36-fold), GMDS (1.17-fold), FBP1 (1.20-fold), FBP2 (2.46-fold), and FUT3 (1.18-fold) (Figure 1(d)). Evidences have shown that fructose-1,6-bisphosphatase-2 (FBP2), a key enzyme for gluconeogenesis, was decreased in various human cells, such as cervical cancer and soft tissue sarcomas [18, 24, 25]. Upregulation of FBP2 could inhibit sarcoma cell and tumor growth *in vitro* and *in vivo* [18]. However, the role of FBP2 in OSCC remains unclear. Thus, FBP2 was selected for the subsequently experiments.

### 3.2. The Methylation of FBP2 Promoter Was Induced during the Progression of OSCC

To investigate the mechanism by which FBP2 was downregulated in OSCC, MSP was performed. As shown in Supplementary Figure 3A, the methylation level of FBP2 is significantly higher in OSCC cells, compared with that in MSK-Leuk1 cells. In addition, 5-Aza significantly decreased the methylation level of FBP2 in OSCC cells (Supplementary Figure 3B), and the expression of FBP2 in OSCC cells was notably upregulated in the presence of 5-Aza (Supplementary Figure 3C). In summary, the methylation of FBP2 promoter was induced during the progression of OSCC.

### 3.3. Overexpression of FBP2 Inhibited the Proliferation and Migration of OSCC Cells

**Figure 2:**
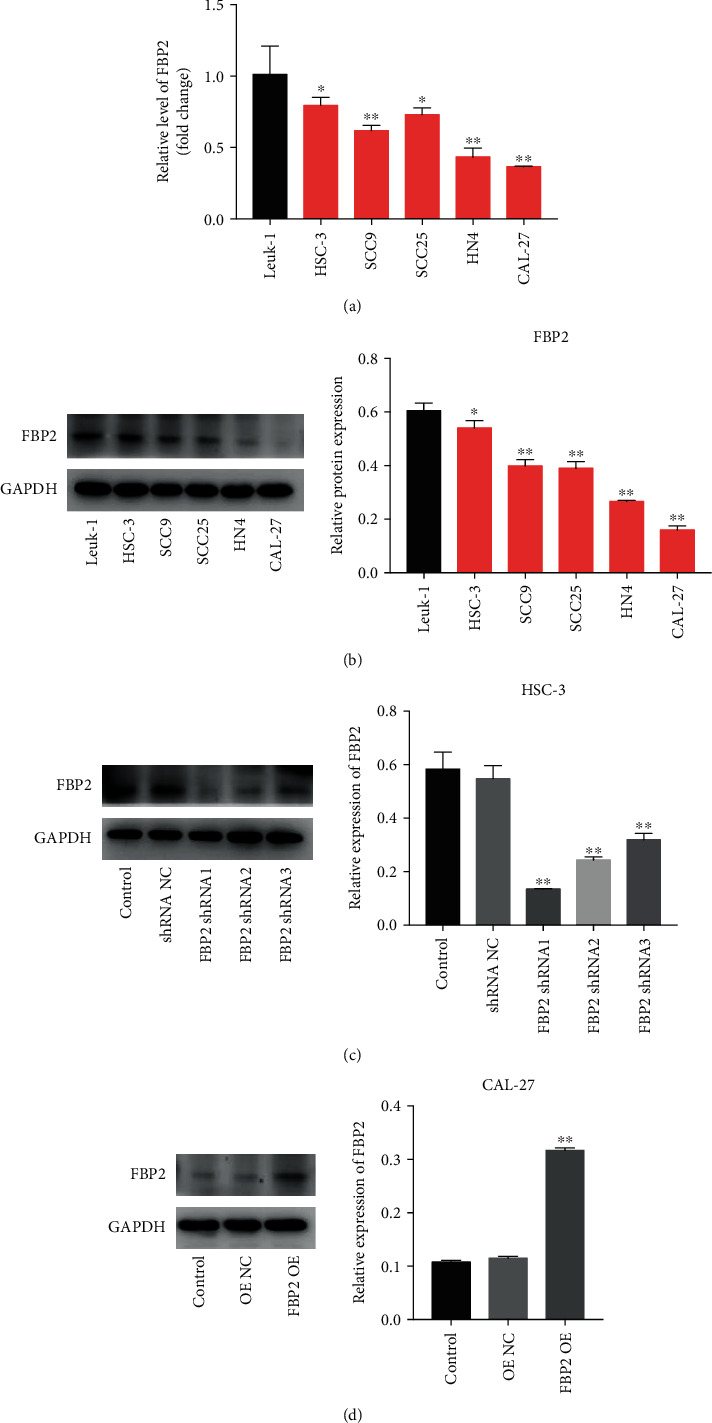
FBP2 is downregulated in OSCC cells. (a) RT-qPCR analysis of FBP2 levels in HSC-3, SCC9, SCC25, HN4, and CAL-27 cells. (b) Western blot analysis of FBP2 expressions in HSC-3, SCC9, SCC25, HN4, and CAL-27 cells. ∗*P* < 0.05, ∗∗*P* < 0.01 vs. Leuk-1 group. (c) Western blot analysis of FBP2 levels in HSC-3 cells transfected with FBP2 shRNA1, FBP2 shRNA2, and FBP2 shRNA3. ∗∗*P* < 0.01 vs. shRNA NC group. (d) FBP2 levels in CAL-27 cells transfected with FBP2 OE were detected using western blot assay. ∗∗*P* < 0.01 vs. OE NC group.

**Figure 3:**
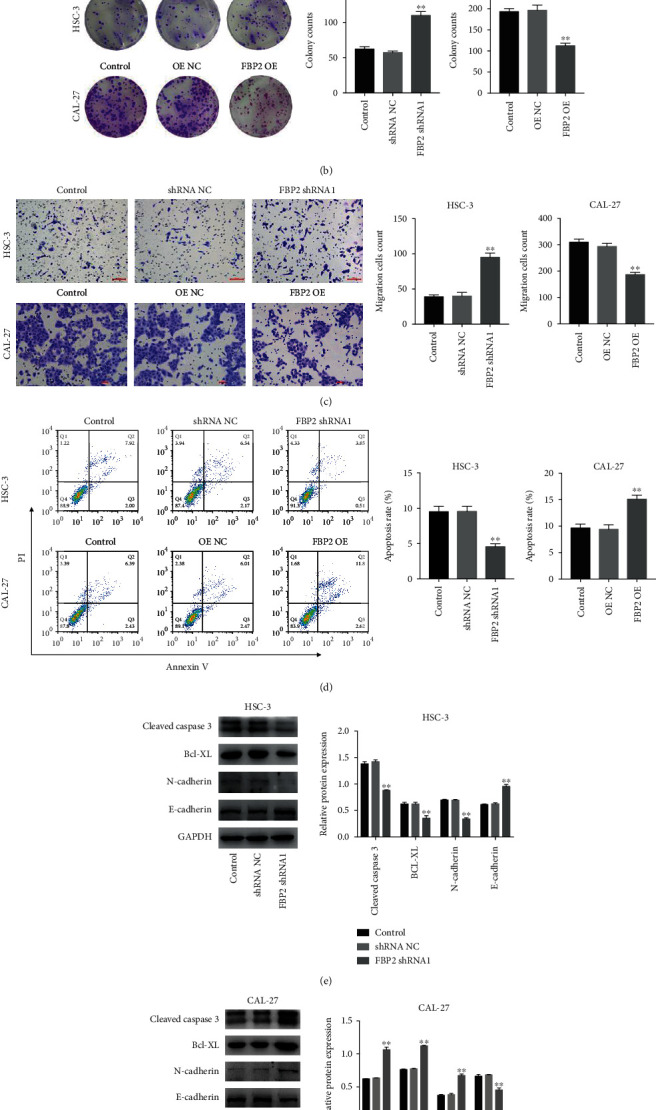
Overexpression of FBP2 inhibited the proliferation, migration and induced the apoptosis of OSCC cells. HSC-3 cells were transfected with FBP2 shRNA1, and CAL-27 cells were transfected with FBP2 OE. (a) CCK-8 assay was used to measure cell viability. (b) Colony formation assay was applied to determine cell proliferation. (c) Cell migration was detected using transwell migration assay. (d) Cell apoptosis was determined using flow cytometry assay. (e, f) Western blot analysis of cleaved caspase 3, BCL-XL, N-cadherin, and E-cadherin levels in transfected HSC-3 and CAL-27 cells. ∗∗*P* < 0.01 vs. shRNA NC or OE NC group.

To explore the role of FBP2 in OSCC cells, we detected FBP levels in Leuk1 cells, and five OSCC cell lines HSC-3, SCC9, SCC25, HN4, and CAL-27, by using RT-qPCR and western blot assays. As indicated in Figures 2(a) and 2(b), the mRNA and protein expression levels of FBP2 are markedly lower in HSC-3, SCC9, SCC25, HN4, and CAL-27 cells compared with Leuk1 cells, respectively. To determine the function of FBP2 in OSCC, we established HSC-3 cells with FBP2 stable knockdown and overexpressed FBP2 in CAL-27 cells. As shown in Figure 2(c), the expression of FBP2 is notably decreased in HSC-3 cells following infection with FBP2 shRNA1. In addition, the expression of FBP2 was significantly increased in CAL-27 cells after transfected with FBP2 OE plasmids (Figure 2(d)). Moreover, the results of CCK-8, colony formation, wound healing, transwell migration, and flow cytometry assays showed that downregulation of FBP2 markedly promoted the viability, proliferation, and migration and inhibited the apoptosis of HSC-3 cells (Figures 3(a)–3(d), Supplementary Figures 4A and 4B). In contrast, upregulation of FBP2 notably inhibited the viability, proliferation, and migration and triggered the apoptosis of CAL-27 cells (Figures 3(a)–3(d), Supplementary Figures 4A and 4B). Meanwhile, downregulation of FBP2 significantly reduced the expressions of cleaved caspase 3, BCL-XL, N-cadherin, and increased the expression of E-cadherin in HSC-3 cells (Figure 3(e)). In contrast, CAL-27 cells transfected with FBP2 OE exhibited the opposite trend (Figure 3(f)). Collectively, FBP2 could suppress the proliferation and migration of OSCC cells.

### 3.4. Overexpression of FBP2 Inhibited the Glycolysis in OSCC Cells

**Figure 4:**
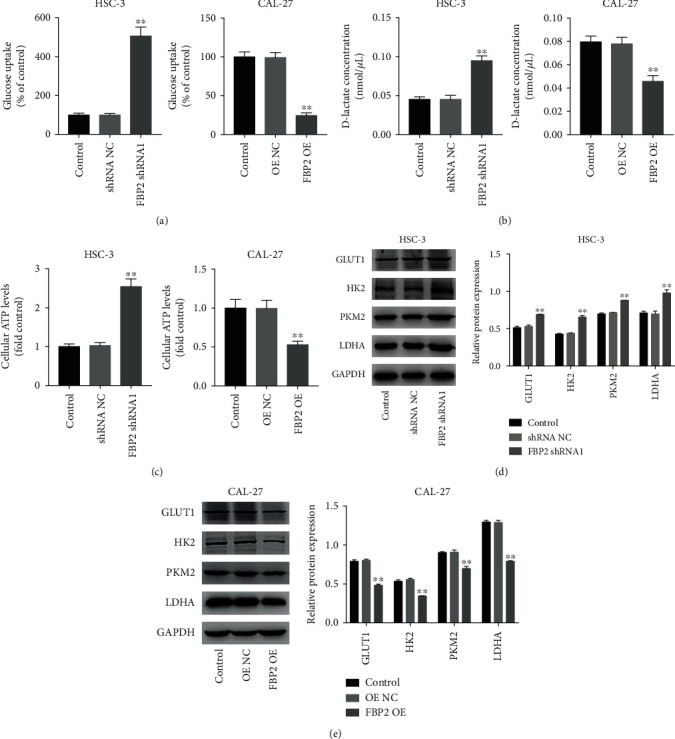
Overexpression of FBP2 inhibited the glycolysis in OSCC cells. HSC-3 cells were transfected with FBP2 shRNA1, and CAL-27 cells were transfected with FBP2 OE. (a) Glucose uptake was measured using a glucose update assay kit. (b) The D-lactate levels in cells was measured using a D-lactate assay kit. (c) ATP content was measured using an ATP assay kit. (d, e) GLUT1, HK2, PKM2, and LDHA levels in HSC-3 and CAL-27 cells were detected using western blot assay. ∗∗*P* < 0.01 vs. shRNA NC or OE NC group.

We further investigated whether FBP2 could change glucose metabolism in OSCC cells. As indicated in Figure 4(a), FBP2 knockdown notably stimulates the glucose uptake in HSC-3 cells, whereas upregulation of FBP2 markedly inhibits the glucose uptake in CAL-27 cells. In addition, the intracellular lactate concentrations were significantly increased in HSC-3 cells transfection with FBP2 shRNA1 (Figure 4(b)). In contrast, CAL-27 cells transfected with FBP2 OE exhibited the opposite results (Figure 4(b)). Next, to determine whether the increased glucose uptake into OSCC cells could result in elevated energy production, we investigated the effect of FBP2 on cellular ATP levels in OSCC cells. As shown in Figure 4(c), downregulation of FBP2 leads to a 2.54-fold increase in ATP content in HSC-3 cells compared with the control group. In contrast, FBP2 overexpression remarkedly decreased the cellular ATP levels in CAL-27 cells (Figure 4(c)). Furthermore, downregulation of FBP2 significantly increased the expressions of the glucose transporter GLUT-1 and glycolytic enzymes HK2, PKM2, and LDHA in HSC-3 cells (Figure 4(d)). Conversely, upregulation of FBP2 in CAL-27 cells resulted in marked decrease in their expressions (Figure 4(e)). To sum up, overexpression of FBP2 could inhibit the glycolysis in OSCC cells.

### 3.5. Downregulation of FBP2 Promoted the Tumorigenesis of HSC-3 Subcutaneous Xenograft *In Vivo* via Enhancing Glycolysis

**Figure 5:**
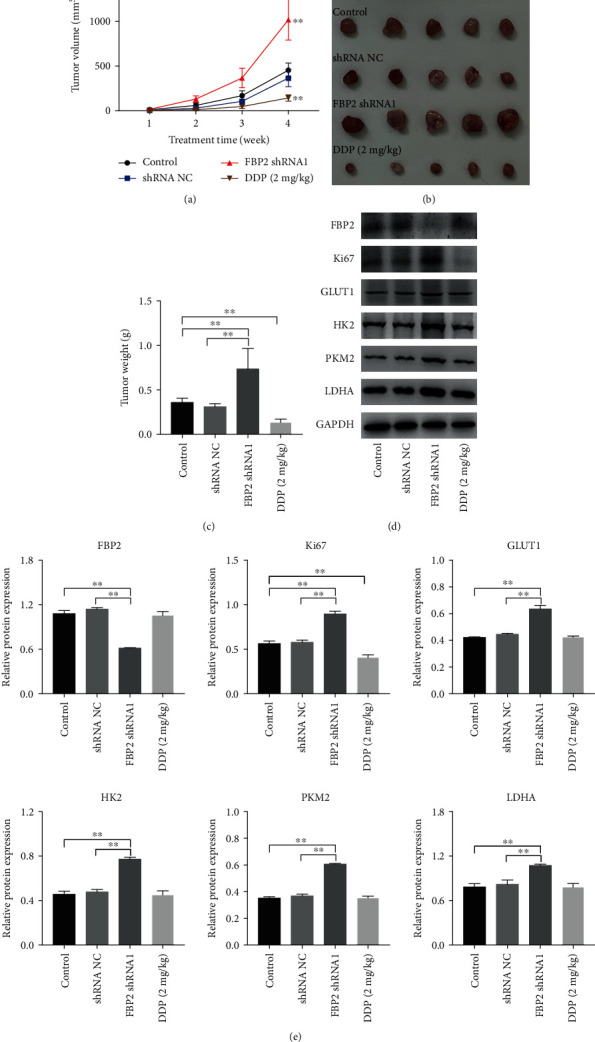
Downregulation of FBP2 promoted the tumorigenesis of HSC-3 subcutaneous xenograft *in vivo* via enhancing glycolysis. (a) Tumor volume was measured. (b) Representative image of the isolated tumors from the xenograft nude-mice model. (c) The tumor weight was measured. (d, e) Western blot analysis of FBP2, Ki67, GLUT1, HK2, PKM2, and LDHA levels in tumor tissues. ∗∗*P* < 0.01.

We further determined the role of FBP2 on OSCC growth *in vivo*, and cisplatin (DDP) was used as positive control. As revealed in Figures 5(a)–5(c), downregulation of FBP2 markedly increases the tumor volume and tumor weight, compared with control or shRNA NC group. Significantly, the tumor volume and weight were decreased by DDP treatment (Figures 5(a)–5(c)). In addition, downregulation of FBP2 notably reduced FBP2 protein expression and upregulated Ki67, GLUT1, HK2, PKM2, and LDHA protein expressions in tumor tissues (Figures 5(d) and 5(e)). Meanwhile, DDP treatment significantly downregulated the expression of Ki67 in tumor tissues, whereas DDP treatment had no effect on the expressions of FBP2, GLUT1, HK2, PKM2, and LDHA in tumor tissues (Figures 5(d) and 5(e)). To sum up, FBP2 knockdown could promote the tumorigenesis of HSC-3 subcutaneous xenograft *in vivo* via enhancing glycolysis.

### 3.6. Overexpression of FBP2 Inhibited Glycolysis in OSCC Cells through Downregulating c-Myc

**Figure 6:**
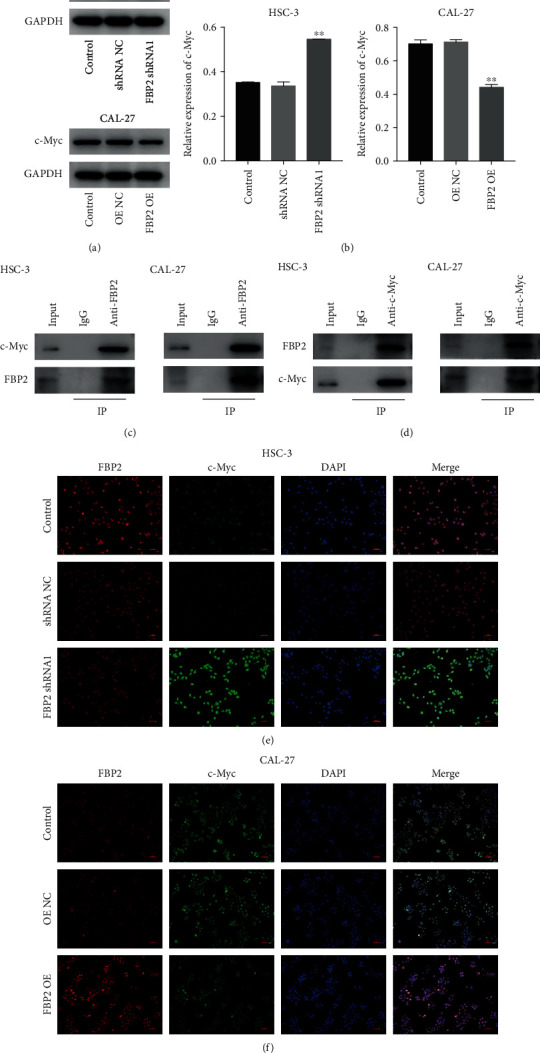
FBP2 binds with c-Myc in the nucleus. (a, b) HSC-3 cells were transfected with FBP2 shRNA1, and CAL-27 cells were transfected with FBP2 OE. Western blot analysis of c-Myc levels in HSC-3 and CAL-27 cells. ∗∗*P* < 0.01 vs. shRNA NC or OE NC group. (c, d) Co-IP was performed to determine the interaction between FBP2 and c-Myc. (e, f) IF images of HSC-3 and CAL-27 cells showed that FBP2 (red) co-localized with c-Myc (green) in the nucleus.

It has been shown that c-Myc plays an important role in the regulation of glucose metabolism, via transcriptionally activating GLUT1, HK2, PKM2, and LDHA [23, 26]. Thus, we then sought to investigate whether FBP2 could affect the expression of c-Myc in OSCC cells. As shown in Supplementary Figure 5A, c-Myc is predicted to be bound with FBP2, and the level of c-Myc is negatively correlated with FBP2 in OSCC tissues (Supplementary Figure 5B). Downregulation of FBP2 notably increased the expression of c-Myc in HSC-3 cells, whereas overexpression of FBP2 decreased the expression of c-Myc in CAL-27 cells (Figures 6(a) and 6(b)). However, whether FBP2 interacted with c-Myc in OSCC cells remains to be further verified. To determine whether FBP2 could bind with c-Myc, co-IP assay was performed in HSC-3 and CAL-27 cells transfected with c-Myc or FBP2. As revealed in Figures 6(c) and 6(d), FBP2 could bind to the c-Myc in HSC-3 and CAL-27 cells. Moreover, the results of IF analysis showed that FBP2 and c-Myc were largely distributed in the nucleus (Figures 6(e) and 6(f)). These data showed that FBP2 could bind with c-Myc in the cell nucleus.

**Figure 7:**
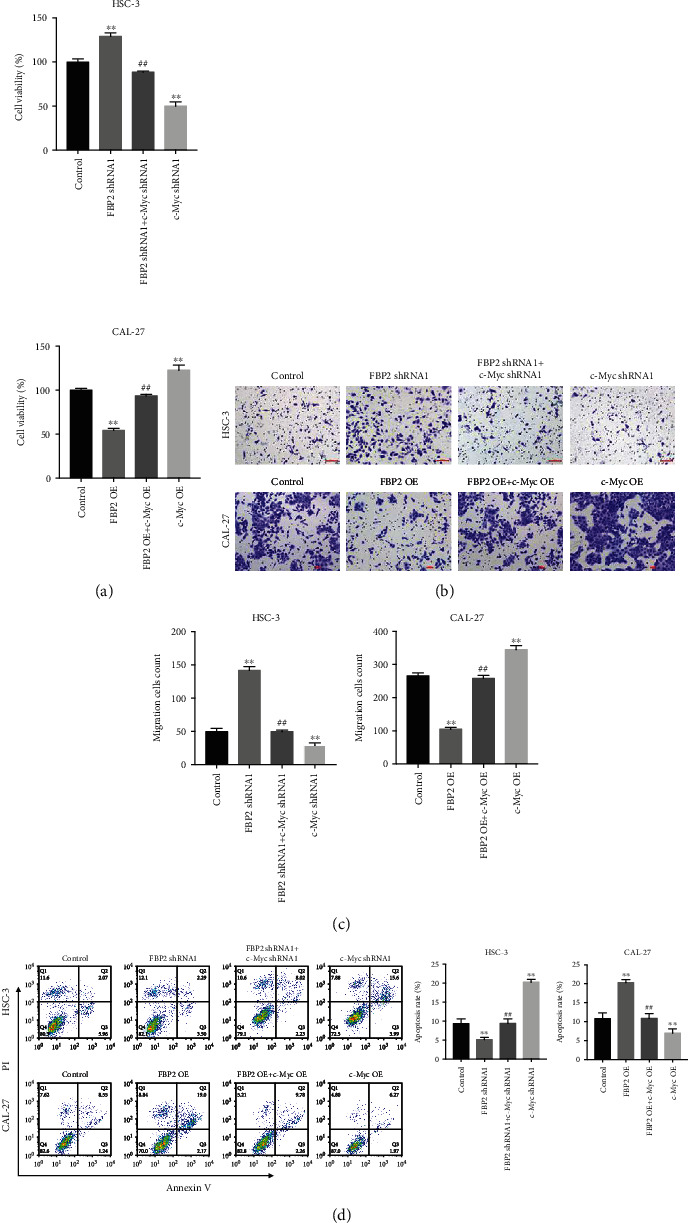
Overexpression of FBP2 inhibited the viability, proliferation, migration in OSCC cells through downregulating c-Myc. HSC-3 cells were transfected with FBP2 shRNA1 or/and c-Myc shRNA1, and CAL-27 cells were transfected with FBP2 OE or/and c-Myc OE. (a) CCK-8 assay was used to measure cell viability. (b) Colony formation assay was performed to determine cell proliferation. (c) Cell migration was detected using transwell migration assay. (d) Cell apoptosis was determined using flow cytometry assay. ∗∗*P* < 0.01 vs. control group; ##*P* < 0.01 vs. FBP2 shRNA1 or OE NC group.

Meanwhile, the expression of FBP2 in OSCC cells was significantly downregulated by FBP2 shRNA but upregulated by FBP2 OE (Supplementary Figure 6A). Consistently, the level of c-Myc in OSCC cells was notably decreased by c-Myc shRNA but increased by c-Myc OE (Supplementary Figure 6B). In addition, FBP2 negatively regulated the level of c-Myc (Supplementary Figure 6B), and the effect of FBP2 shRNA/OE on c-Myc level was partially reversed by c-Myc shRNA/OE (Supplementary Figure 6B). Furthermore, FBP2 knockdown markedly promoted the viability, proliferation, and migration and inhibited the apoptosis of HSC-3 cells; however, c-Myc inhibition largely abolished the promoting effect of FBP2 knockdown on the growth of HSC-3 cells (Figures 7(a)–7(d), and Supplementary 7A). Meanwhile, inhibitory effects of FBP2 overexpression on the viability, proliferation, and migration were reversed by c-Myc overexpression (Figures 7(a)–7(c) and Supplementary 7B). These data showed that overexpression of FBP2 could inhibit the viability, proliferation, and migration in OSCC cells through downregulating c-Myc.

**Figure 8:**
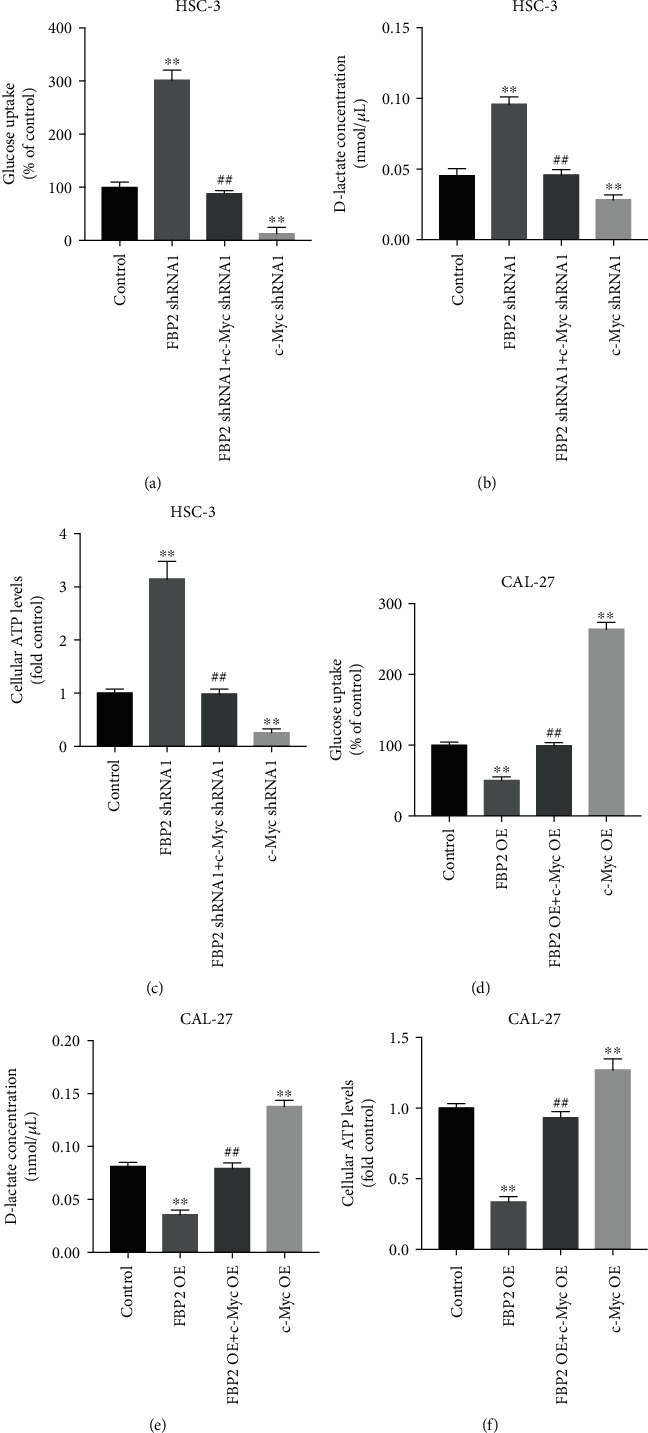
Overexpression of FBP2 inhibited glycolysis in OSCC cells through downregulating c-Myc. HSC-3 cells were transfected with FBP2 shRNA1 or/and c-Myc shRNA1, and CAL-27 cells were transfected with FBP2 OE or/and c-Myc OE. (a) Glucose uptake, (b) the D-lactate levels, and (c) ATP contents were measured in HSC-3 cells. (d) Glucose uptake, (e) the D-lactate levels, and (f) ATP contents were measured in CAL-27 cells. ∗∗*P* < 0.01 vs. control group; ##*P* < 0.01 vs. FBP2 shRNA1 or OE NC group.

Significantly, downregulation of FBP2 stimulated the glucose uptake in HSC-3 cells, followed by elevation of intracellular lactate concentrations and cellular ATP levels, whereas these phenomena were largely reversed by c-Myc knockdown (Figures 8(a)–8(c)). In contrast, overexpression of FBP2 inhibited the glucose uptake in CAL-27 cells, followed by reduced intracellular lactate concentrations and cellular ATP levels, whereas these phenomenon were largely reversed by c-Myc overexpression (Figures 8(d)–8(f)). Collectively, FBP2 could inhibit glycolysis in OSCC cells through downregulating c-Myc.

## 4. Discussion

In this study, we found that FBP2 is downregulated in OSCC tissues. In addition, overexpression of FBP2 significantly inhibited the proliferation, migration and glycolysis of OSCC cells via downregulation of c-Myc. Mechanistically, the ability of FBP2 to inactivate c-Myc signaling contributes to decreased glycolysis, leading to the inhibition of OSCC progression.

Deregulated glucose metabolism is considered as a cancer hallmark that contributes to tumor progression [27, 28]. In contrast to healthy cells, most cancer cells rely on aerobic glycolysis as the main energy to sustain uncontrolled tumor cell proliferation, i.e., the Warburg effect [29]. In addition, gluconeogenesis is a reverse process of glycolysis and can antagonize aerobic glycolysis in cancer [30]. Glucose metabolism is balanced by the anabolic (e.g., gluconeogenesis) and catabolic (e.g., glycolysis) processes [31]. FBP2 is a key enzyme for gluconeogenesis, which plays important roles in energy and glucose metabolism [24]. Thus, re-expression of FBP2 might antagonize glycolysis and then decrease glucose uptake by cancer cells. In the present study, we found that overexpression of FBP2 significantly suppressed the glucose uptake in OSCC cells, followed by reduced intracellular lactate concentrations and cellular ATP levels. In addition, overexpression of FBP2 decreased the expressions of glycolytic genes of GLUT1, LDHA, HK2, and PKM2 in OSCC cells, which resulted in reduced glycolytic activity. Collectively, overexpression of FBP2 could inhibit glycolysis in OSCC.

It has been shown that gluconeogenic enzymes such as FBP localize in the nucleus and could affect gene transcription [18, 32]. Li et al. found that nuclear FBP1 could inhibit transcription factor HIF activity and then decrease its downstream targets (PDK1, LDHA, GLUT1), therefore inhibiting glycolytic activity [33]. In addition, the transcription factor c-Myc plays important roles in the regulation of cellular growth and metabolism [34]. Ectopic c-MYC expression could drive aerobic glycolysis in cancers via the direct upregulation of GLUT1, LDHA, HK2, and PKM2 [35, 36]. In this study, we found that FBP2 directly binds to c-Myc in the nucleus. In addition, overexpression of FBP2 significantly downregulated the expression of c-Myc in OSCC cells, which led to decreased glycolytic activity. In contrast, overexpression of c-Myc reversed FBP2 overexpression-mediated glycolytic inhibition. These data suggested that FBP2 might act as a nuclear c-Myc transcriptional corepressor, which was consistent with the previous study [18]. Collectively, FBP2 could inhibit glycolysis in OSCC cells through downregulating c-Myc.

## 5. Conclusion

In this study, we provided the evidence that FBP2 overexpression could inhibit the migration and invasion and suppressed the glycolysis in OSCC cells by inhibiting c-Myc-mediated glycolysis. Our results may provide a new treatment strategy for OSCC.

## Figures and Tables

**Table 1:** Primer sequences.

Name		Primer sequences (5′–3′)
BHMT	Forward	TGCTGGAGAGATTGTGATTGGA
	Reverse	CTTGTCTTCACTCGCATAGAAGG
GGT6	Forward	AATTCCACGGCCCTGACATC
	Reverse	CCATCAGCATGGCAAAGTAGT
GMDS	Forward	TGCACTATGGCGATCTCACTG
	Reverse	ACTCAGCGAGGTCAAAGGAAA
FBP1	Forward	CGCGCACCTCTATGGCATT
	Reverse	TTCTTCTGACACGAGAACACAC
FBP2	Forward	ACCCGCTACGTTATGGAAAAG
	Reverse	GCCGTCAGCATTGAGTTCAG
CBR3	Forward	TGGACATCGACGACTTGCAG
	Reverse	TGTTGACCAGTACGTTGAGCC
FUT3 c-Myc	Forward	CTGTCCCGCTGTTCAGAGATG
	Reverse Forward Reverse	AGGCGTGACTTAGGGTTGGA GTCAAGAGGCGAACACACAAC TTGGACGGACAGGATGTATGC
GAPDH	Forward	GGAGCGAGATCCCTCCAAAAT
	Reverse	GGCTGTTGTCATACTTCTCATGG

## Data Availability

The datasets used and/or analyzed during the current study are available from the corresponding author on reasonable request.

## Supplementary Materials

Supplementary Materials Supplementary Figure 1 Heat map. (A) Heat maps of DEGs in GSE35261 dataset (testing dataset). (B) Heat maps of DEGs in in-house dataset (validation dataset). Supplementary Figure 2 Volcano plot. (A) Volcano plot of EDGs in OSCC and normal tissues from the GSE35261 dataset (testing dataset). (B) Volcano plot of EDGs in OSCC and normal tissues from the in-house dataset (validation dataset). (C) 142 overlapping DEGs were identified using R language. Supplementary Figure 3 The methylation of FBP2 promoter was induced during the progression of OSCC. (A) The methylation level of FBP2 in LeuK-1 cells was detected by MSP. “U” indicates unmethylated nucleotides. “M” indicates methylated nucleotides. (B) OSCC cells were treated with 5-Aza. The methylation level of FBP2 in OSCC cells was detected by MSP. (C) OSCC cells were treated with 5-Aza. The level of FBP2 in OSCC cells was investigated by RT-qPCR. ∗∗*P* < 0.01 vs. control or LeuK1 group. Supplementary Figure 4 FBP2 inhibited the migration of OSCC cells. (A, B) HSC-3 cells were transfected with FBP2 shRNA1, and CAL-27 cells were transfected with FBP2 OE. Wound-healing assay was performed to evaluate cell migration. ∗∗*P* < 0.01 vs. shRNA NC or OE NC group. Supplementary Figure 5 c-Myc was identified to be the downstream target of FBP2. (A) The downstream target of FBP2 was predicted by Contra V3, JASPAR and TRANSFAC. (B) The level of FBP2 and c-Myc in OSCC or adjacent normal tissues were detected by RT-qPCR. ∗∗*P* < 0.01 vs. normal group. Supplementary Figure 6 FBP2 negatively regulated c-Myc in OSCC cells. (A) The efficiency of FBP2 knockdown or overexpression was tested by western blot. (B) OSCC cells were treated with FBP2 shRNA1, c-Myc shRNA1, or FBP2 shRNA1 + c-Myc shRNA1. The protein level of c-Myc in OSCC cells was detected by western blot. OSCC cells were treated with FBP2 OE, c-Myc OE, or FBP2 OE + c-Myc OE. The protein level of c-Myc in OSCC cells was detected by western blot. ∗∗*P* < 0.01 vs. control group. ##*P* < 0.01 vs. FABP2 shRNA or FABP2 OE group. Supplementary Figure 7 FBP2 inhibited the migration in OSCC cells through downregulating c-Myc. (A, B) HSC-3 cells were transfected with FBP2 shRNA1 or/and c-Myc shRNA1, and CAL-27 cells were transfected with FBP2 OE or/and c-Myc OE. Wound-healing assay was performed to evaluate cell migration. ∗∗*P* < 0.01 vs. control group; ##*P* < 0.01 vs. FBP2 shRNA1 or OE NC group.
