# Supplementary material for: Fructose-1,6-Bisphosphatase 2 Inhibits Oral Squamous Cell Carcinoma Tumorigenesis and Glucose Metabolism via Downregulation of c-Myc
Source: Oxid Med Cell Longev. 2022 May 6;2022:6766787. doi: 10.1155/2022/6766787 (PMC9106462; doi:10.1155/2022/6766787)
Supplement: Supplementary Materials — Supplementary Figure 1 Heat map. (A) Heat maps of DEGs in GSE35261 dataset (testing dataset). (B) Heat maps of DEGs in in-house dataset (validation dataset). Supplementary Figure 2 Volcano plot. (A) Volcano plot of EDGs in OSCC and normal tissues from the GSE35261 dataset (testing dataset). (B) Volcano plot of EDGs in OSCC and normal tissues from the in-house dataset (validation dataset). (C) 142 overlapping DEGs were identified using R language. Supplementary Figure 3 The methylation of FBP2 promoter was induced during the progression of OSCC. (A) The methylation level of FBP2 in LeuK-1 cells was detected by MSP. “U” indicates unmethylated nucleotides. “M” indicates methylated nucleotides. (B) OSCC cells were treated with 5-Aza. The methylation level of FBP2 in OSCC cells was detected by MSP. (C) OSCC cells were treated with 5-Aza. The level of FBP2 in OSCC cells was investigated by RT-qPCR. ∗∗P < 0.01 vs. control or LeuK1 group. Supplementary Figure 4 FBP2 inhibited the migration of OSCC cells. (A, B) HSC-3 cells were transfected with FBP2 shRNA1, and CAL-27 cells were transfected with FBP2 OE. Wound-healing assay was performed to evaluate cell migration. ∗∗P < 0.01 vs. shRNA NC or OE NC group. Supplementary Figure 5 c-Myc was identified to be the downstream target of FBP2. (A) The downstream target of FBP2 was predicted by Contra V3, JASPAR and TRANSFAC. (B) The level of FBP2 and c-Myc in OSCC or adjacent normal tissues were detected by RT-qPCR. ∗∗P < 0.01 vs. normal group. Supplementary Figure 6 FBP2 negatively regulated c-Myc in OSCC cells. (A) The efficiency of FBP2 knockdown or overexpression was tested by western blot. (B) OSCC cells were treated with FBP2 shRNA1, c-Myc shRNA1, or FBP2 shRNA1 + c-Myc shRNA1. The protein level of c-Myc in OSCC cells was detected by western blot. OSCC cells were treated with FBP2 OE, c-Myc OE, or FBP2 OE + c-Myc OE. The protein level of c-Myc in OSCC cells was detected by western blot. ∗∗P < 0.01 vs. control group. ##P < 0.01 [file 6766787.f1.docx]

**Supplementary Figure 1 Heatmap. (A)** Heat maps of DEGs in GSE35261 dataset (Testing dataset). **(B)** Heat maps of DEGs in in-house dataset (Validation dataset).

**
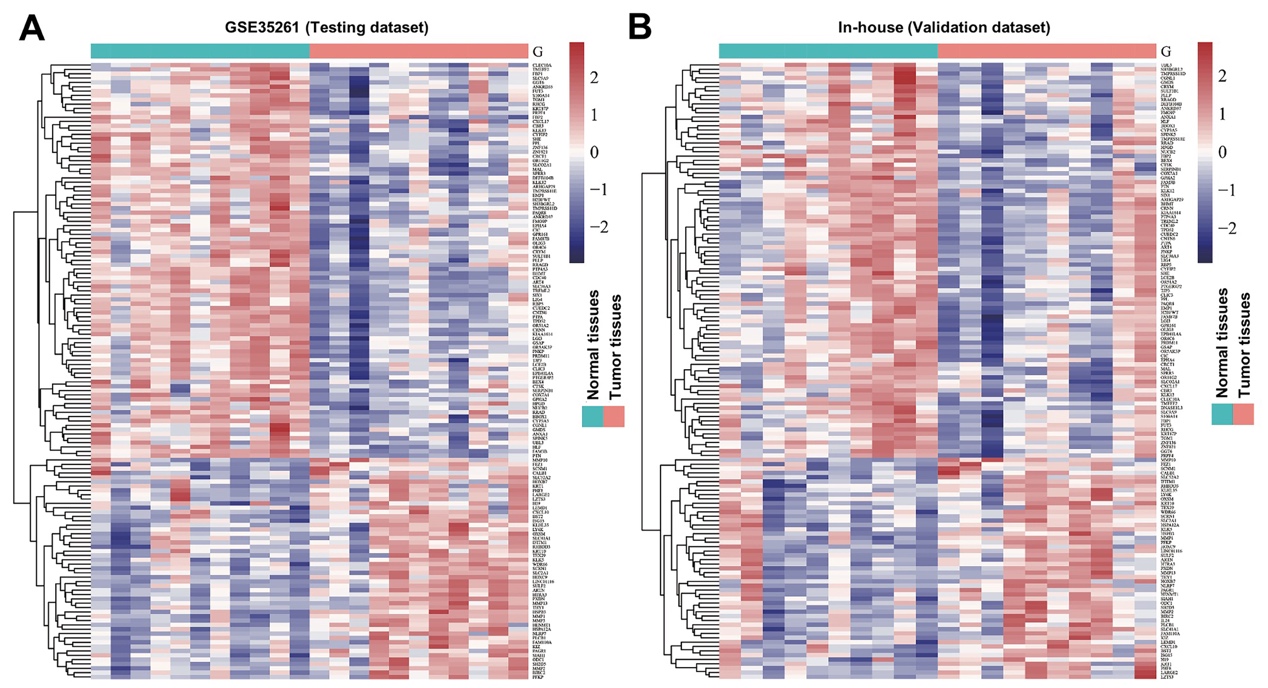
**

**Supplementary Figure 2 Volcano plot. (A)** Volcano plot of EDGs in OSCC and normal tissues from the GSE35261 dataset (Testing dataset). **(B)** Volcano plot of EDGs in OSCC and normal tissues from the in-house dataset (Validation dataset). **(C)** 142 overlapping DEGs were identified using R language.

**
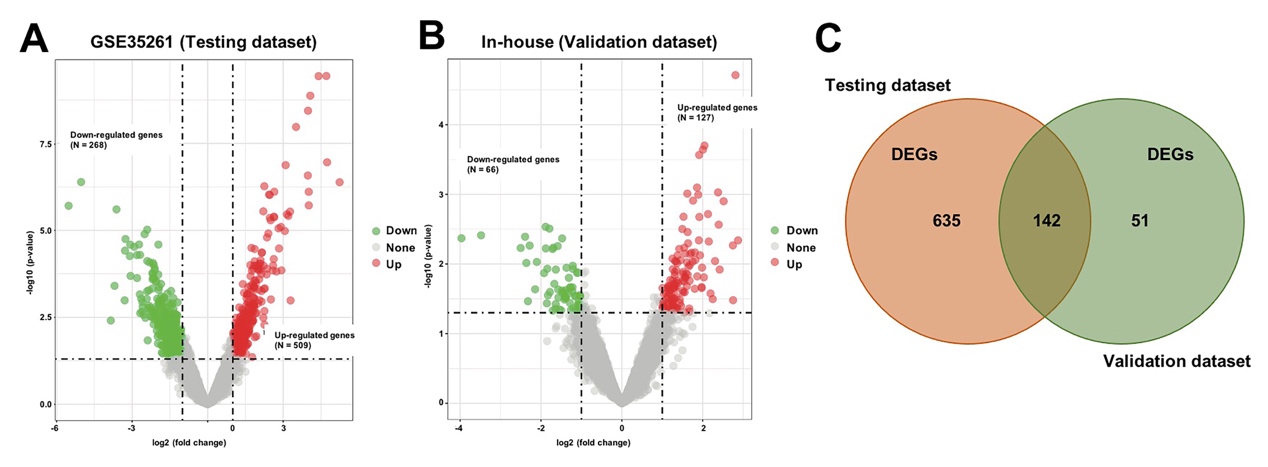
**

**Supplementary Figure 3 The methylation of FBP2 promoter was induced during the progression of OSCC. (A)** The methylation level of FBP2 in LeuK-1 cells was detected by MSP. ‘U’ indicates unmethylated nucleotides. ‘M’ indicates methylated nucleotides. **(B)** OSCC cells were treated with 5-Aza. The methylation level of FBP2 in OSCC cells was detected by MSP. **(C)** OSCC cells were treated with 5-Aza. The level of FBP2 in OSCC cells was investigated by RT-qPCR. **P<0.01 vs. control or LeuK1 group.


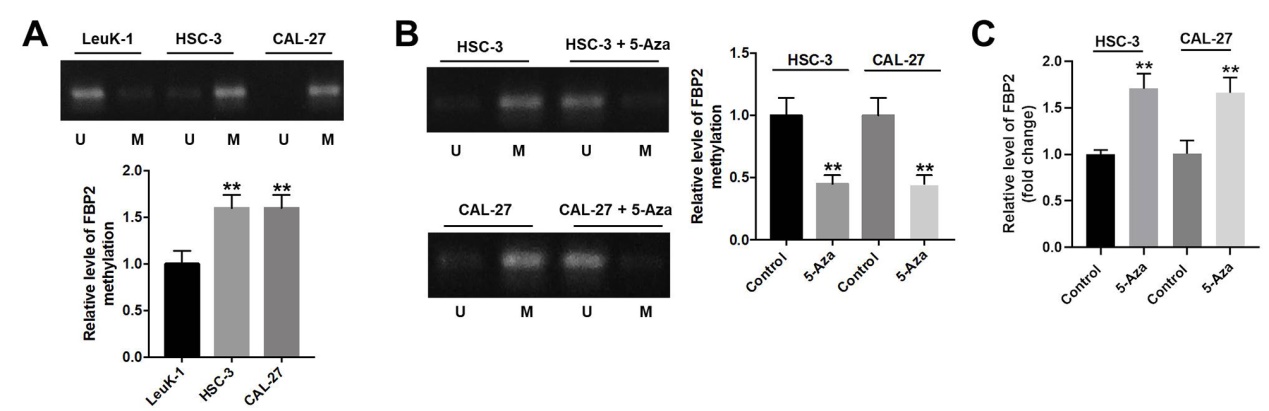


**Supplementary Figure 4 FBP2 inhibited the migration of OSCC cells. (A, B)** HSC-3 cells were transfected with FBP2 shRNA1, and CAL-27 cells were transfected with FBP2 OE. Wound healing assay was performed to evaluate cell migration. **P<0.01 vs. shRNA NC or OE NC group.

**
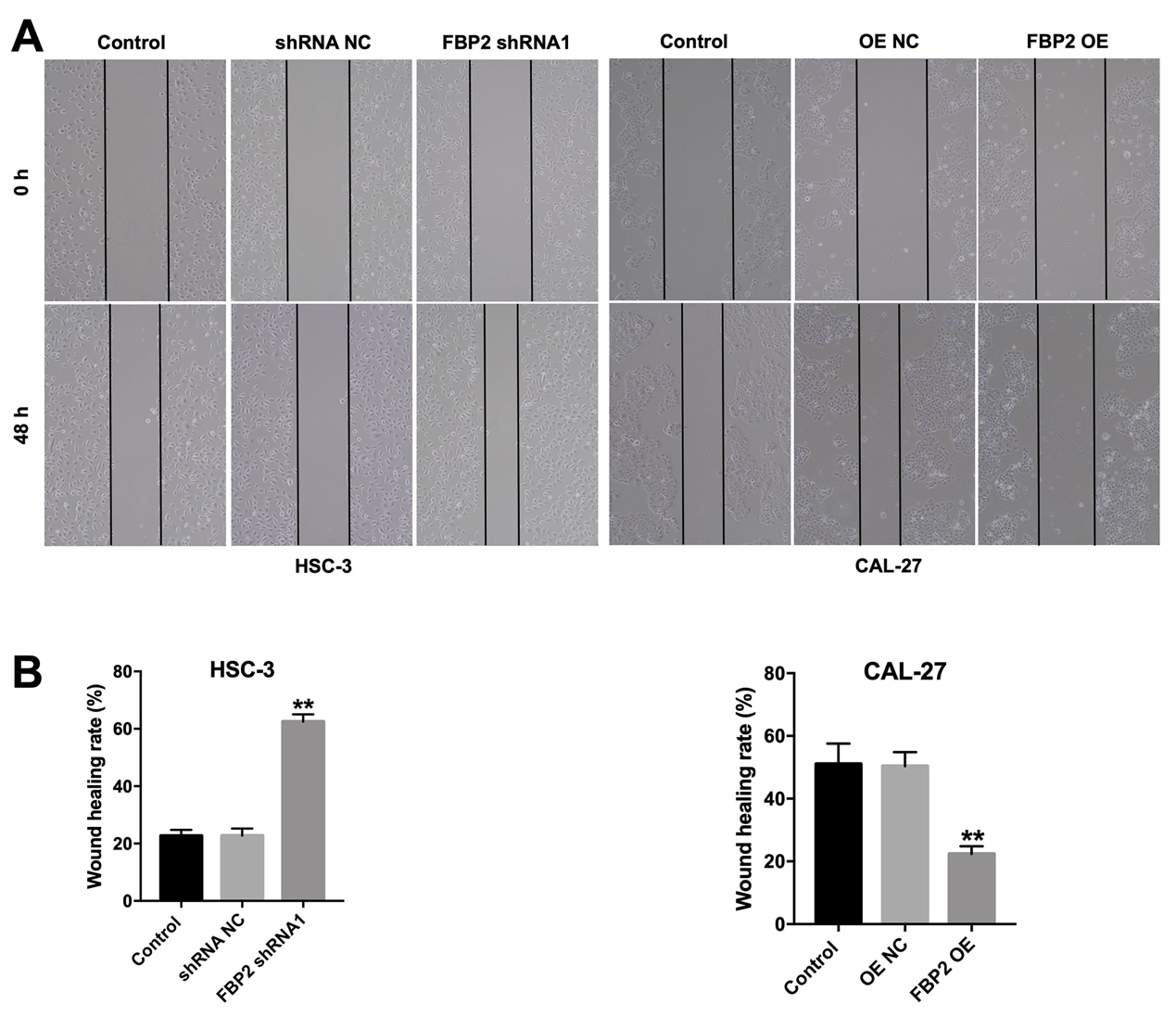
**

**Supplementary Figure 5 c-Myc was identified to be the downstream target of FBP2.** **(A)** The downstream target of FBP2 was predicted by Contra V3, JASPAR and TRANSFAC. **(B)** The level of FBP2 and c-Myc in OSCC or adjacent normal tissues were detected by RT-qPCR. **P<0.01 vs. normal group.

**
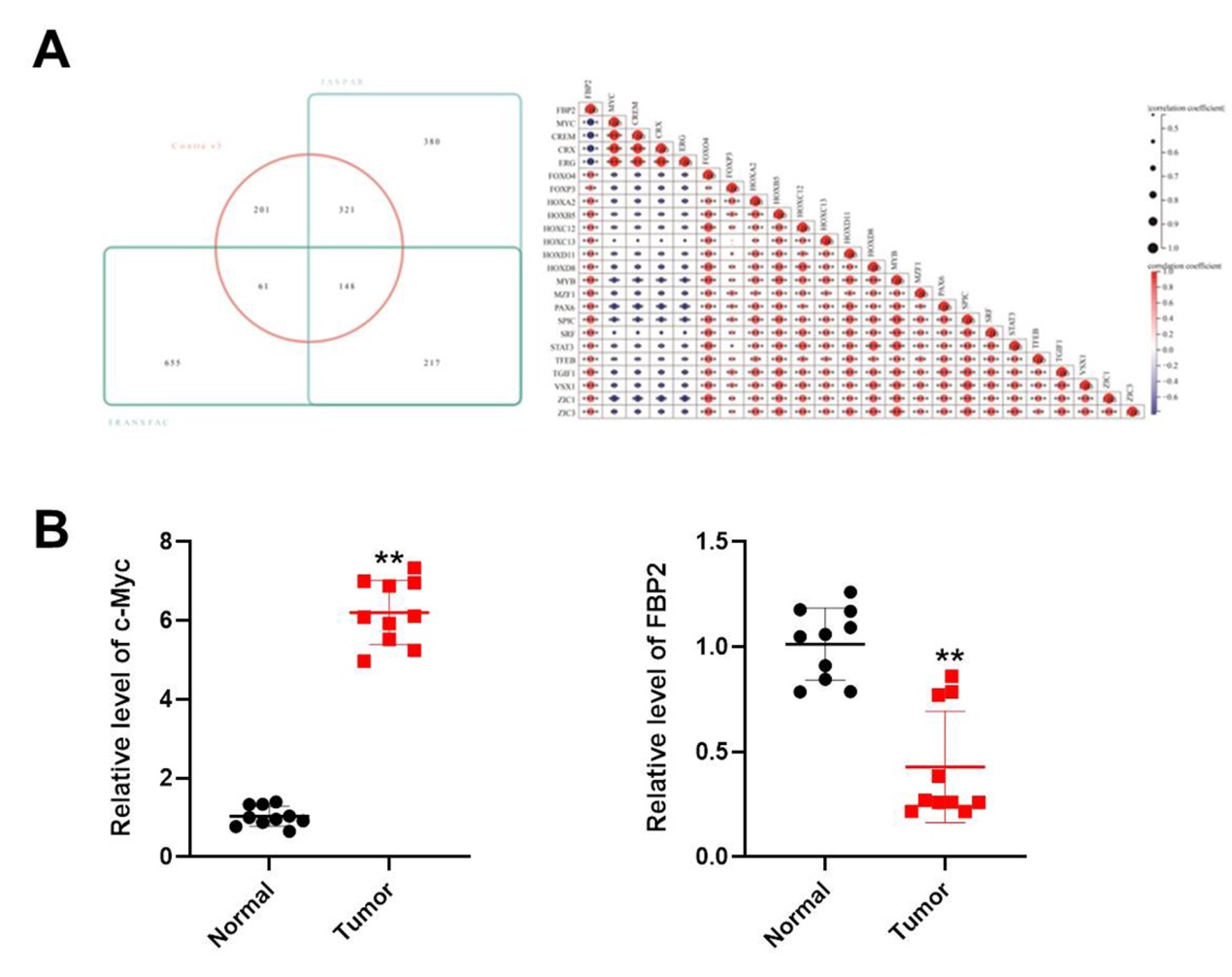
**

**Supplementary Figure 6 FBP2 negatively regulated c-Myc in OSCC cells. (A)** The efficiency of FBP2 knockdown or overexpression was tested by western blot. **(B)** OSCC cells were treated with FBP2 shRNA1, c-Myc shRNA1 or FBP2 shRNA1 + c-Myc shRNA1. The protein level of c-Myc in OSCC cells was detected by western blot. OSCC cells were treated with FBP2 OE, c-Myc OE or FBP2 OE + c-Myc OE. The protein level of c-Myc in OSCC cells was detected by western blot. **P<0.01 vs. control group. ^##^P<0.01 vs. FABP2 shRNA or FABP2 OE group.

**
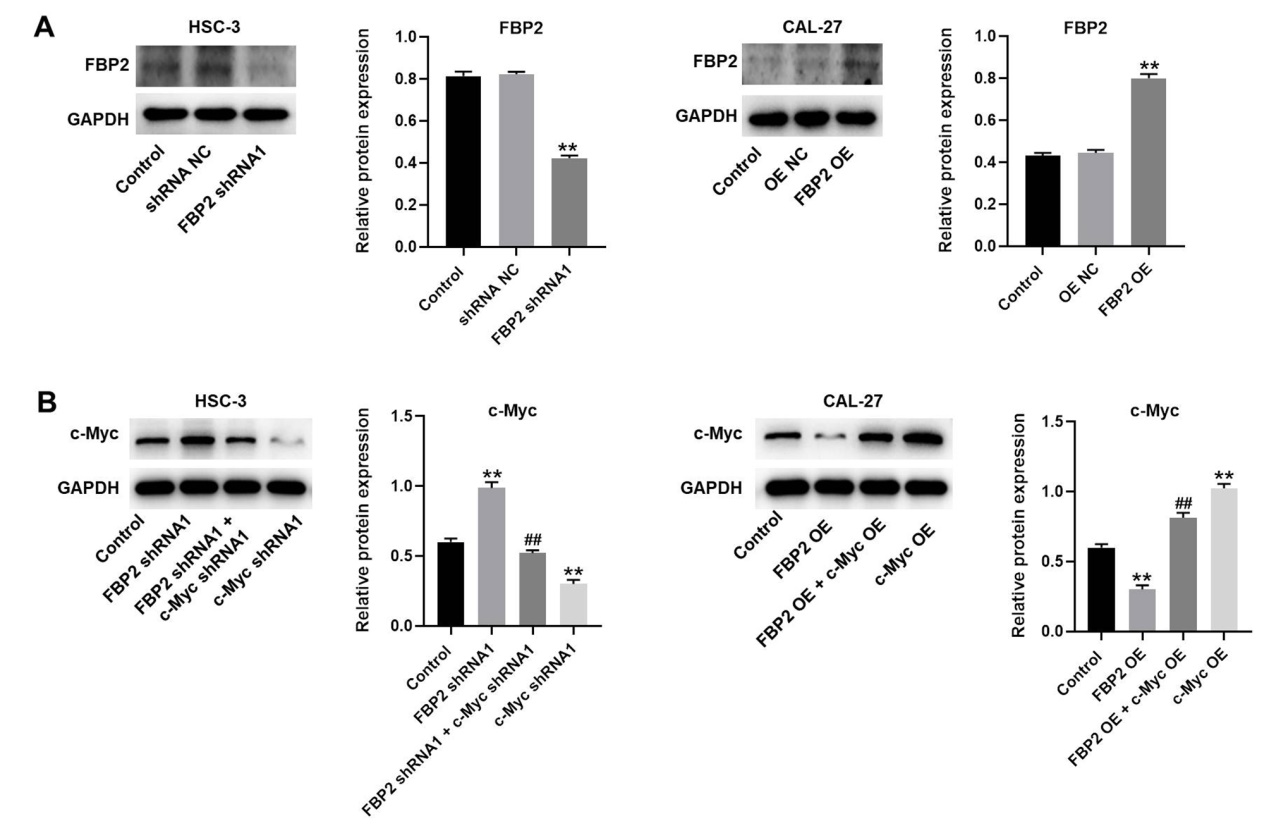
**

**Supplementary Figure 7 FBP2 inhibited the migration in OSCC cells through downregulating c-Myc. (A, B)** HSC-3 cells were transfected with FBP2 shRNA1 or/and c-Myc shRNA1, and CAL-27 cells were transfected with FBP2 OE or/and c-Myc OE. Wound healing assay was performed to evaluate cell migration. **P<0.01 vs. control group; ^##^P<0.01 vs. FBP2 shRNA1 or OE NC group.

**
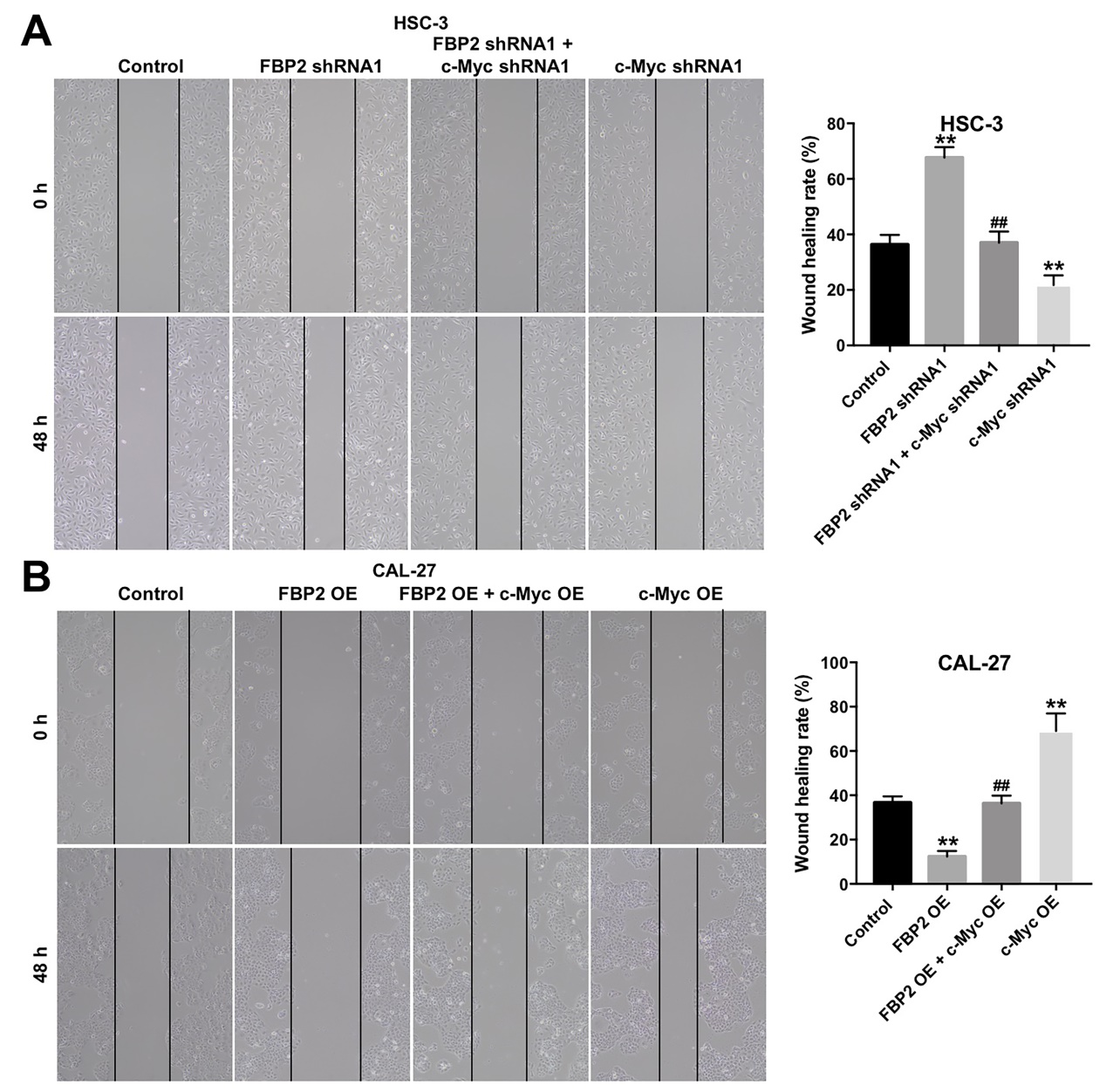
**
